# Functional testicular torsion secondary to an incarcerated inguinal hernia in a 4-month old: complete recovery at 18-hours

**DOI:** 10.1093/jscr/rjab022

**Published:** 2021-02-16

**Authors:** Gregory M Taylor, Christian C Strachan

**Affiliations:** Assistant Professor of Clinical Emergency Medicine, Indiana University School of Medicine, IU Health Ball Memorial Hospital, Muncie, IN, USA; Assistant Professor of Clinical Emergency Medicine and Executive Vice Chair of Clinical Affairs, Indiana University School of Medicine, Muncie, IN, USA

## Abstract

One of the most common urological emergencies encountered in pediatric patients in the emergency department (ED) is the acute scrotum. We present the case of a 4-month-old male that presented to our community ED with scrotal swelling and vomiting of 16-hours duration. He was diagnosed with a functional testicular torsion from an incarcerated inguinal hernia, transferred to a hospital with pediatric urological capabilities and was taken to the operating room ~2 hours later. His hospital course was unremarkable, and he was discharged on day 3, having made a full recovery without any loss of bowel or testicle. There have only been a handful of cases in the literature of a pediatric patient presenting with a functional testicular torsion as a result of spermatic cord compression from an indirect inguinal hernia, with no reported cases of complete salvage at nearly 18 hours since symptom onset.

## INTRODUCTION

A testicular salvage rate of up to 90% has been reported if surgical intervention is performed within the first 6 hours. Identifying an acute scrotum, especially in the neonatal/infant period, can be challenging. Physical exam findings may be subtle, without any signs of trauma. The acute scrotum requires a high index of clinical suspicion in order to potentially save a testicle.

## CASE REPORT

**Figure 1:**
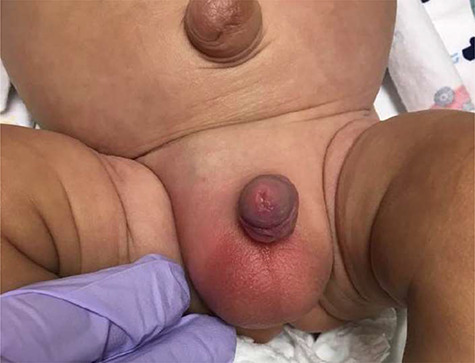
Right scrotum and inguinal region demonstrating erythema, in addition to mottling to the lower abdomen and upper legs bilaterally.

A 4-month-old male presented with his mother to our community emergency department (ED) with the chief complaint of scrotal swelling and vomiting. He had a significant past medical history of oligohydramnios, retinal detachment, seizures, atrial septal defect and a premature vaginal delivery at 23 weeks and 5 days with a resulting prolonged neonatal intensive care stay at an outside hospital. Approximately 16-hours prior to arrival, the mother noticed a small amount of erythema to the scrotum. Since then, the scrotum and inguinal region had been increasing in size, with associated nausea and multiple episodes of vomiting. Vitals on arrival to the ED: afebrile, heart rate of 137 beats/minute, respiratory rate of 30 breaths/minute, weight of 2.637 kg, and 100% on room air. On physical exam, the child appeared ill, lethargic and in distress. Abdominal and genital-urinary exam was notable for a slightly distended abdomen. The patient would cry to palpation of the bilateral lower quadrants and right inguinal region. The right scrotum was firm to the touch. There was associated mottling to the lower abdomen and upper legs bilaterally. There was immediate concern for testicular ischemia/torsion and/or incarcerated/strangulated hernia **(**Fig. 1**).**

**Figure 2:**
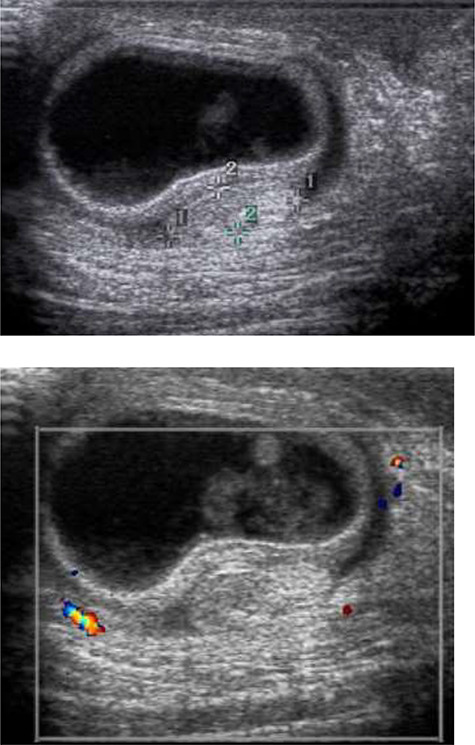
Ultrasound of the scrotum was performed assessing gray scale appearance and color Doppler flow. The right testis and inguinal region demonstrates a right inguinal hernia containing a loop of bowel within the right scrotum with echogenic bowel contents. A hyperechoic ovoid structured, favored to represent the right testicle, is seen measuring ~1.0 × 1.0 × 0.4 cm. There is no sonographic evidence of vascular flow with the right testicle.

While awaiting transfer to a hospital with pediatric surgical capabilities, intravenous fluid resuscitation was initiated. Ultrasound of the scrotum was performed assessing gray scale appearance and color Doppler flow. The left testis demonstrates a homogenous echotexture and measured ~1.5 × 0.6 × 1.3 cm in the longitudinal, anterior/posterior and transverse dimension. Vascular flow is demonstrated on color Doppler images with arterial and venous waveforms. The right testis and inguinal region demonstrated a right inguinal hernia containing a loop of bowel within the right scrotum with echogenic bowel contents. A hyperechoic ovoid structured, favored to represent the right testicle, is seen measuring ~1.0 × 1.0 × 0.4 cm. There is no sonographic evidence of vascular flow to the right testicle **(**Fig. 2**).** This combination of findings is favored to represent testicular vascular compromise and probably infarction secondary to spermatic cord compression from the right inguinal hernia. There is additional concern that this right inguinal hernia is incarcerated. The outside hospital was updated regarding our ultrasound findings, with pediatric general surgery and urology awaiting arrival of the patient for immediate surgical intervention at ~18 hours post symptom onset. The patient’s hospital course was unremarkable, his testicle and all bowel were preserved. He was transferred to the intensive care unit after surgery on high-flow and was discharged home 3-days later. At his 1-month follow-up appointment, the patient is doing well, and his mother denies any concerns.

## DISCUSSION

One of the most common urological emergencies encountered in pediatric patients in the ED is the acute scrotum. Potential causes include:

- Scrotal trauma (ranging from a hematoma of the tunica vaginalis, intratesticular hematoma, disruption of the tunica albuginea, to a testicular rupture),
- Incarcerated inguinal hernia,
- Orchitis/epididymitis,
- Referred pain from a retrocecal appendicitis,
- Testicular torsion, and
- Testicular infarction/ischemia [1].

An inguinal hernia within the pediatric population is considered a common condition, with an incidence ranging between 0.8–4.4% in those patients born term, increasing to 30% in those born premature [5]. These hernias can give rise to not just testicular ischemia, but also intestinal obstruction and ovarian ischemia. There have only been a handful of cases in the literature of a pediatric patient presenting with a functional testicular torsion as a result of spermatic cord compression from an indirect inguinal hernia, with no reported cases of complete salvage at 18 hours post symptom onset [1, 3]. Infants < 6 months have been shown to be at the highest risk for testicular ischemia [5]. A testicular salvage rate of up to 90% has been reported if surgical intervention is performed within the first 6 hours; however, this declines to < 10% at 24 hours [2, 7]. In adults, collateral vessels allow protection from testicular ischemia in the setting of an inguinal hernia. In neonates/infants, the vascular supply is incompletely developed and delicate, and lacks the rich collateral network of vessels. This leads to a higher likelihood of testicular ischemia from an incarcerated inguinal hernia within this age group [6].

Testicular ischemia begins with the hernia causing venous obstruction and increased pressure within a narrow and relatively rigid inguinal ring [5, 6]. This ultimately results in venous thrombosis, hemorrhage and arterial insufficiency, with the end result being infarction of the unilateral testicle [4, 6].

In our patient, he had a functional testicular torsion, meaning, an incarcerated inguinal hernia causing absent vascular flow to the testicle. While twisting of the vessels is the most common reason for testicular torsion, vessel compression, as can be seen with an incarcerated/strangulated inguinal hernia, can result in ischemia/infarction. Identifying an acute scrotum, especially in the neonatal/infant period can be challenging. Physical exam findings may be subtle, without any signs of trauma. The clinician needs to maintain a high index of clinical suspicion in order to potentially save a testicle.

## Data Availability

Data sharing is not applicable to this article as no datasets were generated or analyzed during the current study.
